# 7-difluoromethoxyl-5,4′-di-n-octyl genistein inhibits ovarian cancer stem cell characteristics through the downregulation of FOXM1

**DOI:** 10.3892/ol.2014.2080

**Published:** 2014-04-22

**Authors:** YING-XIA NING, QING-XIU LI, KAI-QUN REN, MEI-FANG QUAN, JIAN-GUO CAO

**Affiliations:** 1 Department of Gynecology and Obstetrics, The First Affiliated Hospital of Guangzhou Medical University, Guangzhou, Guangdong 510120, P.R. China; 2 Laboratory of Medicine Engineering, Medical College, Hunan Normal University, Changsha, Hunan 410013, P.R. China

**Keywords:** ovarian cancer, cancer stem cells, therapeutic action, 7-difluoromethoxyl-5, 4′-di-n-octylgenistein

## Abstract

7-Difluoromethoxyl-5,4′-di-n-octylgenistein (DFOG) is a novel synthetic genistein analogue that possesses anti-cancer activity in a variety of cancers, including ovarian cancer. The objective of the present study was to investigate whether DFOG inhibits the self-renewal capacity of ovarian cancer stem-like cells (OCSLCs) and to identify its potential mechanism of action. It was found that the sphere-forming cells (SFCs) of the SKOV3 cell line exhibited a self-renewal capacity and high tumorigenicity, indicating that they possessed the properties of ovarian cancer stem cells (OCSCs). It was also shown for the first time that DFOG preferentially inhibited proliferation, self-renewal capacity and expression of stem cell markers [cluster of differentiation (CD)133, CD44 and aldehyde dehydrogenase 1 (ALDH1)] in the SFCs derived from the SKOV3 cells. These effects were accompanied by the downregulation of forkhead box M1 (FOXM1) expression. Overexpression of FOXM1 rescued the DFOG-induced downregulation of FOXM1, CD133, CD44 and ALDH1 protein expression. It also inhibited the self-renewal capacity of the SFCs derived from the SKOV3 cells. Thus, DFOG appears to inhibit the characteristics of OCSLCs by downregulating FOXM1 expression.

## Introduction

Ovarian cancer is the most lethal of all the gynecological malignancies. Initial treatment is associated with a 70% response rate, but the majority of patients eventually relapse due to chemoresistance (1). The identification of novel molecular markers that target chemoresistant disease would, therefore, assist in improving the outcome of ovarian cancer therapy.

Cancer stem cells (CSCs) are known to represent a small sub-population of highly malignant cells (2). These cells have an increased resistance to apoptosis and DNA damage, and are therefore more likely to be resistant to chemotherapy and be associated with relapse (3). A previous study in acute myelogenous leukemia has provided compelling evidence for the existence of CSCs (4). Other studies have characterized CSCs in other types of tumors, including brain, breast, colon, pancreas, prostate and ovarian cancer (5–8).

Self-renewal and lineage capacity are hallmarks of all stem cells (5). Various methods have been developed to capitalize on these characteristics in order to obtain cancer stem cells. Evaluating the capacity of cancer cells to grow as multi-cellular spheroids in stem-condition culture systems is one such method (6). Using this method, ovarian CSCs (OCSCs) have been obtained from patients with ascites (7). The most common use of cell surface markers to identify OCSCs in ovarian cancer involves the use of cluster of differentiation (CD)133^+^ cell populations (8).

Forkhead box M1 (FOXM1) regulates transcriptional genes that are necessary for cell cycle progression and cell survival (9,10). The FOXM1 transcription factor is upregulated in the majority of human cancers, indicating that it may participate in the initiation of human carcinogenesis (11). A previous study has shown that FOXM1 expands the pool of human epithelial stem/progenitor cells (11). It has also been reported that the overexpression of FOXM1 leads to epithelial-mesenchymal transition (EMT) and a CSC phenotype in pancreatic cancer cells (12). FOXM1 may therefore be a novel target for therapeutic agents that target CSCs (11,12).

Genistein (4′,5,7-trihydroxyisoflavone; GEN) has a similar chemical structure to that of estrogen, but more extensive biological activities (13,14). GEN has been shown to inhibit tyrosine kinase and reduce cancer cell proliferation *in vivo* and *in vitro* without causing toxicity to non-cancerous cells (15). A study by Wang *et al* has previously shown that FOXM1 activation is inhibited by GEN in pancreatic cancer cells, resulting in apoptotic cell death (16). The low absorption of GEN in the intestine and its rapid metabolic elimination resulting from the hydroxyl groups at the C-5, C-7 and C-4′ positions allows GEN to bind to glucuronic and sulfuric acid. This reduces its bioavailability and bioactivity *in vivo* and restricts its clinical usefulness (17). Studies performed at the Laboratory of Medicine Engineering, Medical College, Hunan Normal University (Changsha, China) have demonstrated that a novel synthetic GEN analogue, 7-difluoromethoxyl-5,4′-di-n-octylgenistein (DFOG), induces cell apoptotic death in ovarian and gastric cancer cells by inactivating FOXM1 (17,18). GEN also has the potential to attenuate FOXM1-mediated cell growth, migration and invasion, the acquisition of an EMT phenotype, and CSC self-renewal capacity in pancreatic cancer cells (12). The present study investigated whether DFOG attenuates the characteristics of OCSCs by inactivating FOXM1.

## Materials and methods

### Cell lines and sphere culture

Human ovarian cell lines, SKOV3 and A2780, were obtained from the Cell Bank of the Chinese Academy of Sciences (Shanghai, China). The cells were maintained as a monolayer in high glucose Dulbecco’s modified Eagle’s medium (DMEM) supplemented with 10% fetal bovine serum, 100 IU/ml penicillin G and 100 μg/ml streptomycin (Life Technologies, Shanghai, China) at 37°C in a humidified 5% CO_2_ incubator.

For the sphere-forming culture, the cells were collected and washed to remove the serum, prior to being suspended in serum-free DMEM/F12 supplemented with 100 IU/ml penicillin, 100 μg/ml streptomycin, 20 ng/ml human recombinant epidermal growth factor, 10 ng/ml human recombinant basic fibroblast growth factor, 2% B27 supplement without vitamin A and 1% N2 supplement (Invitrogen, Carlsbad, CA, USA). The cells were subsequently cultured in ultra-low attachment 6-well plates (Corning Inc., Corning, NY, USA) at a density of ≤2,000 cells/well.

### Sphere passage and sphere formation assay

The spheres were collected by gentle centrifugation (500 × g for 5 min), dissociated with trypsin-EDTA and mechanically disrupted with a pipette. The resulting single cells were centrifuged (500 × g for 5 min) to remove the enzyme, and re-suspended in serum-free medium where they were allowed to re-form spheres. The spheres were passaged every 8 days until they reached a diameter of 100 μm. Dissociated single sphere-forming cells (SFCs) were diluted to a density of 500 cells/ml. The diluted cell suspension was plated onto ultra-low attachment 96-well plates at 2 μl/well (Corning Inc.). Serum-free medium (150 μl) was then added. Wells with only one cell were marked and observed every day.

### In vivo tumorigenicity experiments

Four-week-old BALB/c-nu male mice (Shanghai Laboratory Animal Center, Chinese Academy of Sciences, Shanghai, China) were housed and maintained in accordance with the Institutional Guidelines of Hunan Normal University (Changsha, Hunan, China). The study was approved by the ethics committee of Hunan Normal University. The SKOV3 parental cells and the third passages of the SFCs were used in the tumorigenicity experiments. Trypan blue staining was used to assess cell viability. Various numbers of viable single cells in serum-free DMEM/Matrigel (1:1; BD Biosciences, Shanghai, China) were subcutaneously injected into the mice using a 100-μl microsyringe [Sangon Biotech (Shanghai) Co., Ltd., Shanghai, China]. The mice were humanely sacrificed 8 weeks after the injection, and the tumors were harvested for further examination.

### MTT assay

The SFCs from the SKOV3 cell line and the parental cells were seeded in 96-well plates (precoated with Matrigel) at a density of 5,000 cells per well. The cells were exposed to increasing concentrations of DFOG. After 48 h, MTT reagent (Sigma-Aldrich, St. Louis, MO, USA) was added to each well according to the manufacturer’s instructions. Absorbance was measured at 570 nm.

### Plasmids and transfection

The FOXM1 cDNA plasmid was purchased from OriGene Technologies Inc. (Rockville, MD, USA). The SFCs derived from the SKOV3 cell line were transfected with cDNA using Lipofectamine 2000 (Life Technologies), as previously described (17).

### Western blot analysis

Western blot analysis was carried out, as previously described (18). Monoclonal mouse anti-FOXM1, monoclonal mouse anti-CD133 and monoclonal mouse anti-CD44 (Cell Signaling Technology, Inc., Danvers, MA, USA), as well as monoclonal mouse anti-aldehyde dehydrogenase 1 (ALDH1) and monoclonal mouse anti-β-actin (Santa Cruz Biotechnology, Inc., Santa Cruz, CA, USA) antibodies were used as primary antibodies. The cells were lysed by being incubated in lysis buffer for 20 min at 4°C. The protein concentration was determined using the Bio-Rad assay system (Bio-Rad, Hercules, CA, USA). Total proteins were fractionated using SDS-PAGE and transferred onto a polyvinylidene fluoride membrane (Millipore, Billerica, MA, USA). Signals were detected using an Enhanced Chemiluminescence Advance western blot analysis system (Amersham Pharmacia Biotech Inc., Piscataway, NJ, USA).

### Statistical analysis

Statistical analysis and database management was undertaken using SPSS version 15.0 software (SPSS, Inc., Chicago, IL, USA). Data are represented as the mean ± standard deviation. Multiple group comparisons were made using one-way analysis of variance, and pairwise comparisons were performed using the least squares difference method. A two-tailed t-test was used when appropriate. Values of P<0.05 were considered to indicate a statistically significant difference.

## Results

### Characteristics of ovarian cancer stem-like cells (OCSLCs) in the SKOV3 cell line

**Figure 1:**
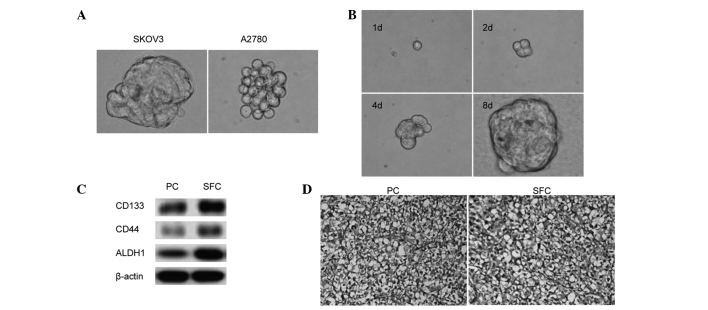
Sphere-forming cells (SFCs) of the SKOV3 cell line highly enriched ovarian cancer stem cells (OCSCs). (A) Human ovarian cancer SKOV3 and A2780 cell lines formed spheres in the stem cell culture system. The volume of SFCs in the SKOV3 cell line was much higher than that of the A2780 cell line (magnification, ×400). (B) Single SFCs from the SKOV3 cell line formed spheres on days 1, 2, 4, and 8, respectively. (C) Western blot analysis showing that cluster of differentiation (CD)133, CD44 and aldehyde dehydrogenase 1 (ALDH1) were highly expressed in the SFCs from the SKOV3 cell line compared with the parental cells. (D) H&E staining showing that the histological features of the xenografted tumors in the SFCs were similar to those from the parental SKOV3 cells (PC; magnification, ×100).

Ovarian cells were plated in stem cell-conditioned culture medium in 6-well plates at a density of 2,000 cells/well, which allowed the formation of discrete colonies. Under these conditions, the cells grew as non-adherent, three-dimensional sphere clusters. Fig. 1A shows the anchorage-independent spheres that formed in the SKOV3 and A2780 cells. The spheres were passaged after 8 days, when they had reached ~50 μm in diameter. The SKOV3 and A2780 spheres were serially passaged for >12 generations, indicating their self-renewal capability *in vitro*. To corroborate the finding that a sphere could be generated from a single cell, single SKOV3 cells were plated on a 96-well plate and the wells with one cell were visualized everyday. Fig. 1B shows the process by which a single SKOV3 cell formed a sphere.

Next, the ovarian CSC markers, CD133, CD44 and ALDH1, were evaluated using western blot analysis; the results showed enrichment of the CD133^+^, CD44^+^ and ALDH-high populations in the SFCs derived from the SKOV3 cells compared with the parental cells (Fig. 1C).

To confirm that the SFCs from the SKOV3 cells exhibited enhanced tumor-initiating capability, BALB/c-nu mice were transplanted with varying numbers of SKOV3 SFCs. SKOV3 parental cells were used as controls. The results indicated that as few as 1,000 SFCs were sufficient for tumor development, whereas, at least 2×10^5^ parental cells were necessary to consistently generate a tumor in the same model over a longer period of time (Table I). The tumor nodules formed by the SFCs of the SKOV3 cell line displayed similar histology to that observed with the parental cells (Fig. 1D). These findings indicated that the tumorigenic efficacy of the SFCs was higher than that of the parental cells, and that non-adherent tumor spheres from the ovarian cancer SKOV3 cell line cultured in stem cell-conditioned medium possess OCSLC properties.

### DFOG inhibits the proliferation and self-renewal of OCSLCs derived from the SKOV3 cell line

**Figure 2:**
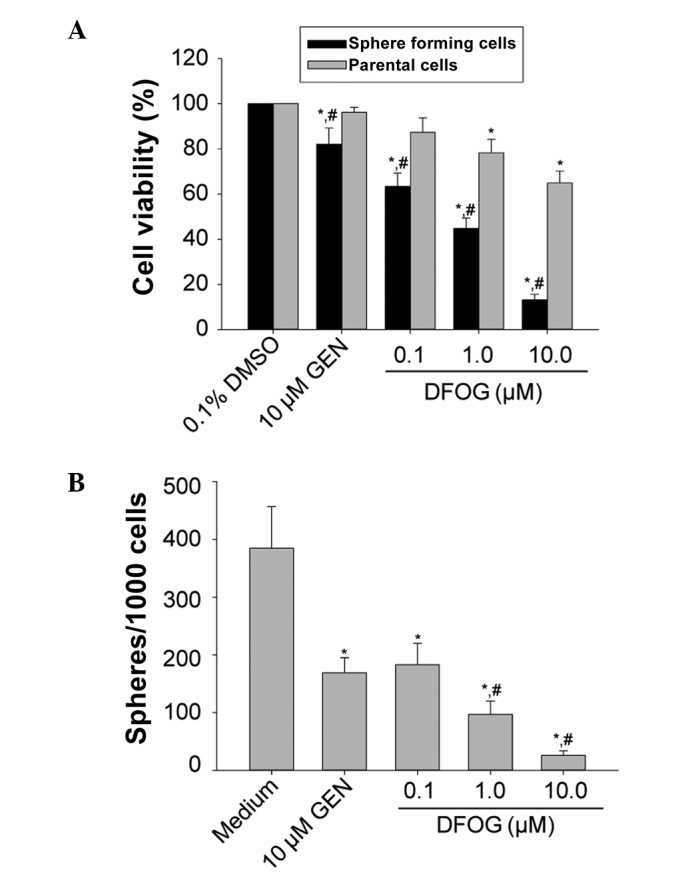
DFOG preferentially inhibits proliferation and self-renewal of OCSLCs derived from SKOV3 cells. (A) DFOG preferentially inhibited the proliferation of the SFCs derived from the SKOV3 cells. (B) Sphere-forming cells (SFCs) were incubated with varying concentrations of DFOG (0.1, 1.0 and 10.0 μmol/l) or dimethyl sulfoxide (DMSO) for 6 days. DFOG inhibited the self-renewal capacity of the SFCs derived from the SKOV3 cells. Data are presented as the mean ± standard deviation (n=3). ^*^P<0.05 vs. 0.1% DMSO; ^#^P<0.05 vs. the parental cells treated with DFOG or 10.0 μmol/l genistein. DFOG, 7-difluoromethoxyl-5,4′-di-n-octylgenistein; OCSLCs, ovarian cancer stem-like cells; GEN, genistein.

It has been reported that CSCs have the characteristics of extensive proliferation, and GEN has been shown to inhibit the proliferative activity of pancreatic cancer stem cells (11). In the present study, the MTT results showed that DFOG (0.1, 1.0 and 10.0 μmol/l) and GEN (10.0 μmol/l) preferentially inhibited the proliferation of the SFCs derived from the SKOV3 cells (P=0.030; Fig. 2A), indicating that DFOG is able to preferentially suppress the proliferative ability of OCSLCs.

DFOG (0.1, 1.0 and 10.0 μmol/l) reduced the number of spheroids formed in the SFCs from the SKOV3 cells in a concentration-dependent manner (P=0.022; Fig. 2B). These results indicate that DFOG can suppress the self-renewal of OCSLCs.

### DFOG downregulates the expression of FOXM1 and CSC markers in OCSLCs derived from the SKOV3 cell line

**Figure 3:**
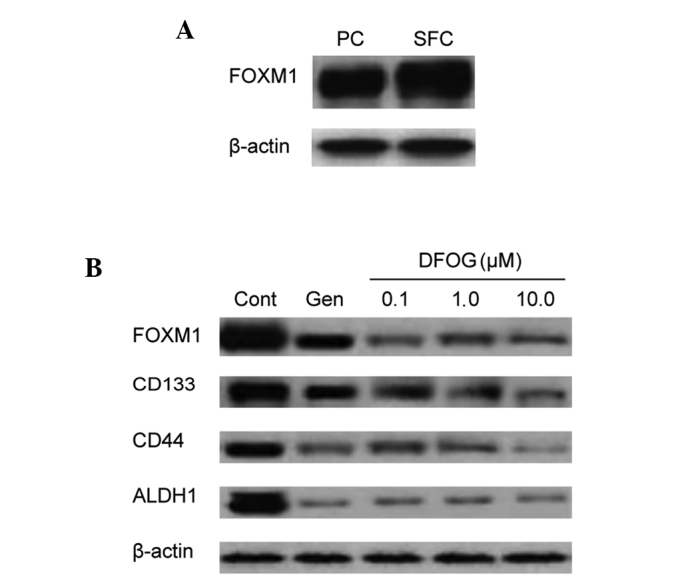
DOFG downregulates FOXM1 and CSC marker expression in OCSLCs derived from SKOV3 cells. (A) FOXM1 is highly expressed in SFCs from the SKOV3 cell line compared with the corresponding parental cells. (B) DFOG downregulated the expression of FOXM1 and CSC markers (CD133, CD44, and ALDH1) in the SFCs derived from the SKOV3 cells. DFOG, 7-difluoromethoxyl-5,4′-di-n-octylgenistein; FOXM1, forkhead box M1; CSCs, cancer stem cells; OCSLCs, ovarian cancer stem-like cells; GEN, genistein; SFCs, sphere-forming cells.

We previously demonstrated the molecular role of FOXM1 in mediating the biological effects of GEN and DFOG in human ovarian cancer cell lines, including the SKOV3 cell line (17). A previous study reported that the overexpression of FOXM1 leads to EMT and the formation of a cancer stem cell phenotype in pancreatic cancer cells (12). Based on these findings, the present study next sought to compare the status of FOXM1 protein expression in the parental cells and SFCs. The results showed that FOXM1 expression was higher in the SFCs compared with the parental cells (Fig. 3A). In addition, FOXM1 expression in the SFCs was downregulated by DFOG (Fig. 3B).

In pancreatic cancer stem cells, the overexpression of FOXM1 has been shown to result in increased sphere-forming capacity and the increased expression of the CSC surface marker, CD44 (12). In the present study, DFOG inhibited the protein expression of CD133, CD44 and ALDH1 in the SFCs (Fig. 3B).

### Overexpression of FOXM1 attenuates the inhibitory effects of DFOG on the expression of CSC markers and the self-renewal in OCSLCs derived from the SKOV3 cell line

**Figure 4:**
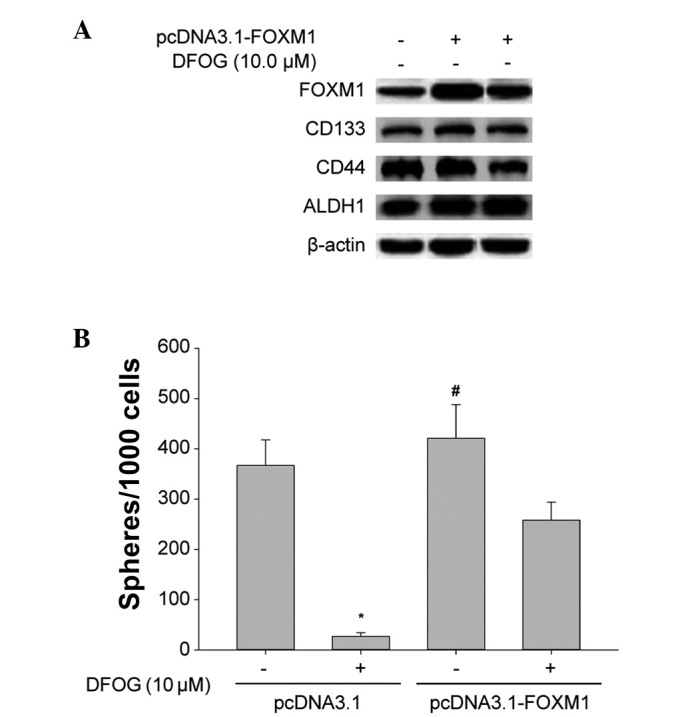
Overexpression of FOXM1 attenuates the inhibitory effect of DFOG on the self-renewal of OCSLCs derived from the SKOV3 cell line. (A) Western blot analysis showing the overexpression of FOXM1 following transfection with plasmid pcDNA3.1-FOXM1 in the SFCs derived from the SKOV3 cells. The overexpression of FOXM1 reduced the DFOG-induced downregulation of FOXM1, CD133, CD44 and ALDH1 expression. (B) The sphere-forming ability of the SFCs derived from the SKOV3 cells exposed to DFOG was partly rescued following overexpression of FOXM1. Data are presented as the mean ± standard deviation (n=3). ^*^P<0.05 vs. 0.1% DMSO. ^#^P<0.05 vs. cells treated with the same concentration of DFOG using pcDNA3.1 transfection. DFOG, 7-difluoromethoxyl-5,4′-di-n-octylgenistein; FOXM1, forkhead box M1; OCSLCs, ovarian cancer stem-like cells; GEN, genistein; SFCs, sphere-forming cells.

Western blot analysis showed that the upregulation of FOXM1 by pcDNA3.1-FOXM1 transfection resulted in the overexpression of FOXM1, CD133, CD44 and ALDH1 proteins in the SFCs from the SKOV3 cells (Fig. 4A). The overexpression of FOXM1 reversed the DFOG-induced downregulation of FOXM1, CD133, CD44 and ALDH1 protein expression levels (Fig. 4A), and reduced the inhibition of self-renewal to a certain extent (Fig. 4B). These results provide mechanistic evidence, indicating that DFOG-inhibited self-renewal is in part due to the inactivation of FOXM1 in OCSLCs derived from SKOV3 cells.

## Discussion

In 1996, undifferentiated multipotent neural cells were first grown and maintained in suspension using a neurosphere assay (19). Following this discovery, anchorage-independent sphere cultures of stem cells then became instrumental in the study of stem cells, including nerve, prostate and mammary stem cells (20). More recently, the functional approach of using sphere formation has been utilized for enriching potential CSC subpopulations when specific CSC markers have not been defined (21). In the present study, it was confirmed that SFCs derived from the SKOV3 cell line possess self-renewal capacity *in vitro* and have greater tumor-initiating capability *in vivo* than the parent cells. It was also found that CD133^+^, CD44^+^ and ALDH-high populations were enriched in tumor spheroids from the SKOV3 cells. These findings indicate that SFCs from SKOV3 cells exhibit the characteristics of OCSCs and are therefore OCSLCs.

CSCs have been identified in many malignant tumor tissues, including ovarian cancer tissues (22–24). CSCs are believed to play critical roles in drug resistance and cancer metastasis, indicating that targeting CSC self-renewal capacity would eliminate the essential cause of tumor recurrence (25). Previous studies at the Laboratory of Medicine Engineering, Medical College, Hunan Normal University have demonstrated that DFOG, a novel synthetic GEN analogue, induces cell apoptotic death in ovarian and gastric cancer cells (17,18). Significantly, the present study shows for the first time that DFOG significantly inhibits the proliferative activity and self-renewal capability of OCSLCs derived from SKOV3 cells.

Numerous studies have demonstrated that GEN inhibits the growth of breast, prostate and pancreatic cancer cells *in vitro* and *in vivo* (17,26). Genes that are critical for the control of cell proliferation, apoptosis, the cell cycle, oncogenesis, transcription regulation and cell signal transduction pathways have been found to be differentially regulated by GEN. These findings are consistent with the apoptosis-inducing effects of GEN being mediated through inactivation of nuclear factor-κB and Akt signaling pathways (26). In our previous studies, it was shown that DFOG-induced cell apoptotic death was mediated by the inactivation of FOXM1 in ovarian and gastric cancer cells (17,18). The present study showed for the first time that the forced overexpression of FOXM1 led to an increased self-renewal capacity of SFCs derived from SKOV3 cells. This was consistent with the increased expression of the CSC cell surface markers, CD133, CD44 and ALDH1, which could be attenuated by exposure to DOFG.

In summary, the present results demonstrated that the forced overexpression of FOXM1 leads to an increased CSC self-renewal capacity, which is inhibited by DFOG and GEN. Therefore, DFOG and GEN may represent a useful way to inhibit CSC activity, making them potentially significant agents for the prevention of recurrence and/or the treatment of human ovarian cancer.

## Figures and Tables

**Table I:** Tumorigenicity experiments with the SFCs derived from the SKOV3 cell line and the parental cells in BALB/c-nu mice.

Cell type	Cell numbers	Incidence, n	Latency, days
Parental cells	5×10^4^	0/4	-
	1×10^5^	0/4	-
	2×10^5^	3/4	35
	5×10^5^	4/4	29
	1×10^6^	4/4	12
CD133^+^ cells	5×10^2^	0/4	-
	1×10^3^	4/4	25
	5×10^3^	4/4	13
	1×10^4^	4/4	9
	5×10^4^	4/4	6

SFCs, sphere-forming cells; CD, cluster of differentiation.
